# Distinct functions and prognostic values of RORs in gastric cancer

**DOI:** 10.1515/med-2020-0406

**Published:** 2020-05-19

**Authors:** Feng Gu, Yuming Liu, Yuan Liu, Shujie Cheng, Jihong Yang, Ming Kang, Wendu Duan, Yan Liu

**Affiliations:** Department of Hepatobiliary, Hospital of HeBei University, Baoding, China; General Hospital of Huabei Petroleum Administration Bureau, Renqiu, China; Beijing Youan Hospital, Capital Medical University, Beijing, China

**Keywords:** gastric cancer, prognosis, proliferation, retinoic acid receptor-related orphan receptors

## Abstract

Retinoic acid receptor-related orphan receptors (RORs) are frequently abnormally expressed in several human malignancies, including gastric cancer (GC). RORs are involved in the development and progression of GC through Wnt signaling pathway receptors and other common receptors. However, the prognostic roles of individual RORs in patients with GC remain elusive. We accessed the prognostic roles of three RORs (RORα, RORβ, and RORγ) through “The Kaplan–Meier plotter” (KM plotter) database in patients with GC. For all patients with GC who were followed for 20 years, the low mRNA expression of all three RORs showed a significant correlation with better outcomes. We further accessed the prognostic value of individual RORs in different clinical pathological features including Lauren classification, clinical stages, pathological grades, HER2 status, and different treatments methods. The RORs demonstrated critical prognostic roles in GC. Expressions of RORs were higher in GC tissues when compared with normal gastric tissues. Moreover, knockdown of RORs significantly inhibited cell proliferation and migration, suggesting an oncogenic role of RORs in human GC. These findings suggest potential roles of RORs as biomarkers for GC prognosis and as oncogenes in GC.

## 1 Introduction

Gastric cancer (GC) is one of the most common malignant tumors of the human body accounting for about one million new cases worldwide each year. GC is ranked as second highest in mortality among malignant tumors, which indicates an obvious threat to human health [1]. The occurrence and development of GC are the result of interactions between multiple genes and various environmental factors. An early diagnosis and timely treatment are needed for a better prognosis of GC [2,3]. Due to novel developments in cell and molecular biology over the last 20 years, our understanding of the pathogenesis of GC has gradually improved. However, the survival rates of patients have not shown much improvement [4]. Molecular analyses of tumor-related molecules, signaling pathways, proteases, and inhibitors are important in the development of therapeutic measures and prognostic predictive measures of GC in clinical practice [5,6].

**Figure 1:**
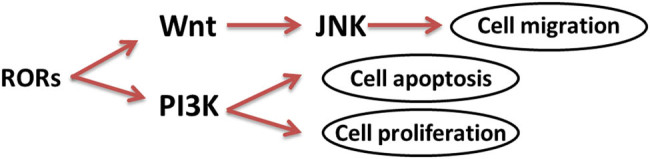
RORs’ signaling pathway.

Retinoic acid receptor-related orphan receptors (RORs) belong to the nuclear receptor super family and consist of three subtypes: RORα (RORA, NR1F1, RZRα), RORβ (RORB, NR1F2, RZRβ), and RORγ (RORC, NR1F3, TOR). RORs play an important role in a series of physiological and pathological processes. These include reproductive development, physiological rhythm regulation, metabolic disorders, inflammation, immune system regulation, and cancer [7,8,9]. Recent studies have shown that RORs is involved in the development and progression of tumors through the Wnt signaling pathway or as common receptors (Figure 1) [9,10,11]. However, the prognostic value of mRNA expression of RORs members in patients with GC remains unclear. Therefore, we sought to elucidate the prognostic value of mRNA expression of RORs in GC to provide a basis for further research.

The online Kaplan–Meier (KM) plotter is capable of assessing the effect of any gene or gene combination on survival in breast, ovarian, lung, and GC using patient samples measured by gene chips or RNA-seq. So far, a number of genes have been identified and validated by the KM plotter in these four cancer types [12,13,14,15,16,17]. This database provided prognostic information and mRNA mapping of 876 patients with GC. In this study, we used the KM plotter database to determine the prognostic roles of mRNA expression of ROR members in patients with GC. In addition, the specific roles of RORs were confirmed in human GC cell line AGS. We predict that RORs potentially serve as novel biomarkers for GC prognosis and oncogenes in GC progression.

## 2 Methods

### 2.1 Survival analysis

The correlation between mRNA expression of RORs and overall survival (OS) was analyzed using the KM plotter database (http://kmplot.com/analysis/). In this online database, gene expression data, relapse free, and OS information are downloaded from GEO (Affymetrix microarrays only), EGA ,and TCGA. The clinical data from 876 patients with GC including gender, perforation history, stage, Lauren classification, HER2 status, pathological grades, and different treatment methods were collected. After entering the three ROR sub-members types into the database (http://kmplot.com/analysis/index.php?p=service&cancer=gastric), data were compared using a Kaplan–Meier survival plot. The hazard ratio (HR) with 95% confidence intervals and log-rank *P* value were also calculated. *P* values of < 0.05 were considered statistically significant. HR is defined as the ratio of the hazard rates corresponding to the conditions described by two levels of an explanatory variable in the survival analysis.

### 2.2 Cell line and transfection

Human GC cell line AGS was obtained from the Type Culture Collection of the Chinese Academy of Sciences. The cells were cultured in dulbecco's modified eagle medium (DMEM) supplemented with 10% fetal bovine serum and incubated at 37℃ with 5% CO_2_. The cells were transfected with siRNA by Lipofectamine 2000 once confluency reached approximately 70%.

### 2.3 Cell Counting Kit-8 (CCK-8) assay

The proliferation of AGS cells transfected with siRNA was confirmed using CCK-8. SiRNA transfected cells were moved into a 96-well plate at 5,000 cells per well. Ten microliters of CCK-8 solutions was added to the well, and the cells were cultured for 1.5 h. Finally, OD values were measured on a microplate reader. Each experiment was performed in triplicate.

### 2.4 Transwell

Transwell assay was performed to evaluate cell migration ability with the transwell chamber (Millipore, USA). SiRNA transfected cells were seeded into the upper chamber. After incubation for 48 h, cells that migrated to the lower chamber were stained with crystal violet for 10 min. Migrated cells were calculated from triplicate determinations.

### 2.5 Immunohistochemistry

GC tissues were stained by immunohistochemistry. Antibodies against the RORs were purchased from Abcam. All samples were fixed in 4% polyformaldehyde and then embedded in paraffin wax. Sections were cut at a thickness of 4 µm. Antigen retrieval was done by heating in a microwave for 12 min. Slides were blocked with goat blocking serum for 40 min followed by incubation with the antibody to h RORs antibody at room temperature for 1 h. The results were observed by a microscope, and all image processing was completed with ImageJ software.

### 2.6 Western bolt

After the transfection for 48 h, total protein was extracted from cells for Western bolt assay. The primary antibodies were purchased from Abcam (RORα, ab60134; RORβ, ab228650; RORγ, ab113434). The relative expressions of RORα, RORβ, and RORγ were calculated by target protein/GAPDH using QUANTITY ONE software.

## 3 Results

### 3.1 High expression of RORs presented with a poor prognosis in human GC

We examined the prognostic value of mRNA expression of RORs via www.kmplot.com. The valid Affymetrix IDs of RORα, RORβ, and RORγ were, respectively, 210479_s_at, 206443_at, and 206419_at. Survival curves were drafted for all patients with GC (*n* = 876), patients with intestinal-type GC (*n* = 320), patients with diffuse-type GC (*n* = 241), and patients with mixed-type GC (*n* = 33).

**Figure 2:**
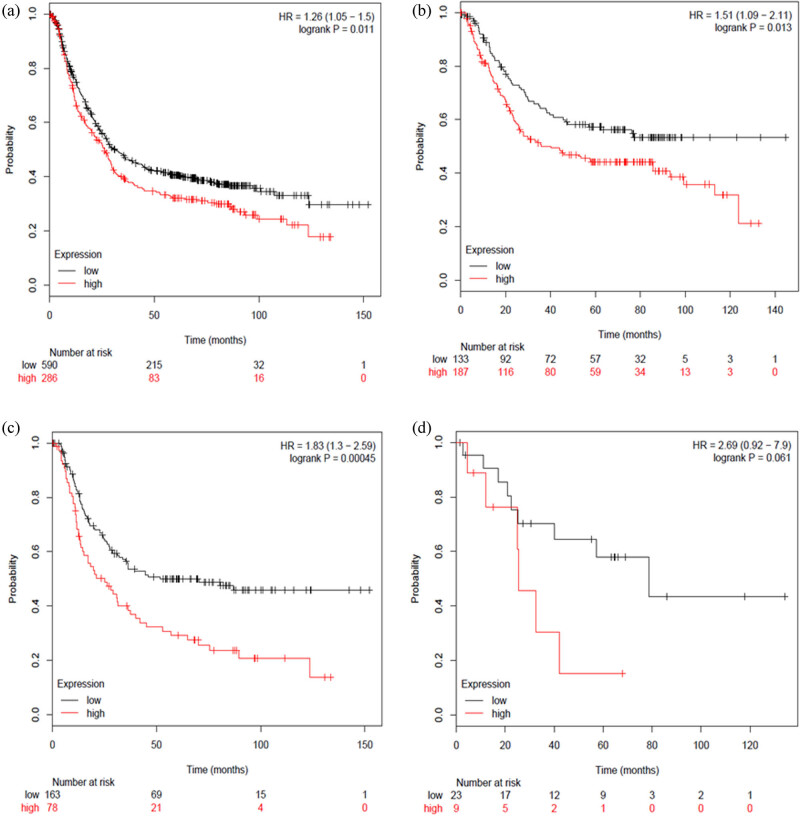
Determination of the prognostic value of RORα mRNA expression using www.kmplot.com. The desired Affymetrix ID is valid: 210479_s_at (RORα). (a) Survival curves were plotted for all patients with GC (*n* = 876). (b) Survival curves were plotted for patients with intestinal-type GC (*n* = 320). (c) Survival curves were plotted for patients with diffuse-type GC (*n* = 241). (d) Survival curves were plotted for patients with mixed-GC (*n* = 33).

First, we assessed the prognostic value of RORα mRNA expression via www.kmplot.com. Low mRNA expression of RORα demonstrated an association with a better prognosis [HR = 1.26 95% CI: (1.05–1.5), *P* = 0.011] (Figure 2a). Low RORα mRNA was also associated with a better prognosis in intestinal-type GC [HR = 1.51, 95% CI: (1.09–2.11), *P* = 0.013] (Figure 2b), as well as in diffuse-type GC [HR = 1.83, 95% CI: (1.3–2.59), *P* = 0.00045] (Figure 2c). However, there was not a significant difference in mixed-type GC [HR = 2.69, 95% CI: (0.92–7.9), *P* = 0.061) (Figure 2d).

**Figure 3:**
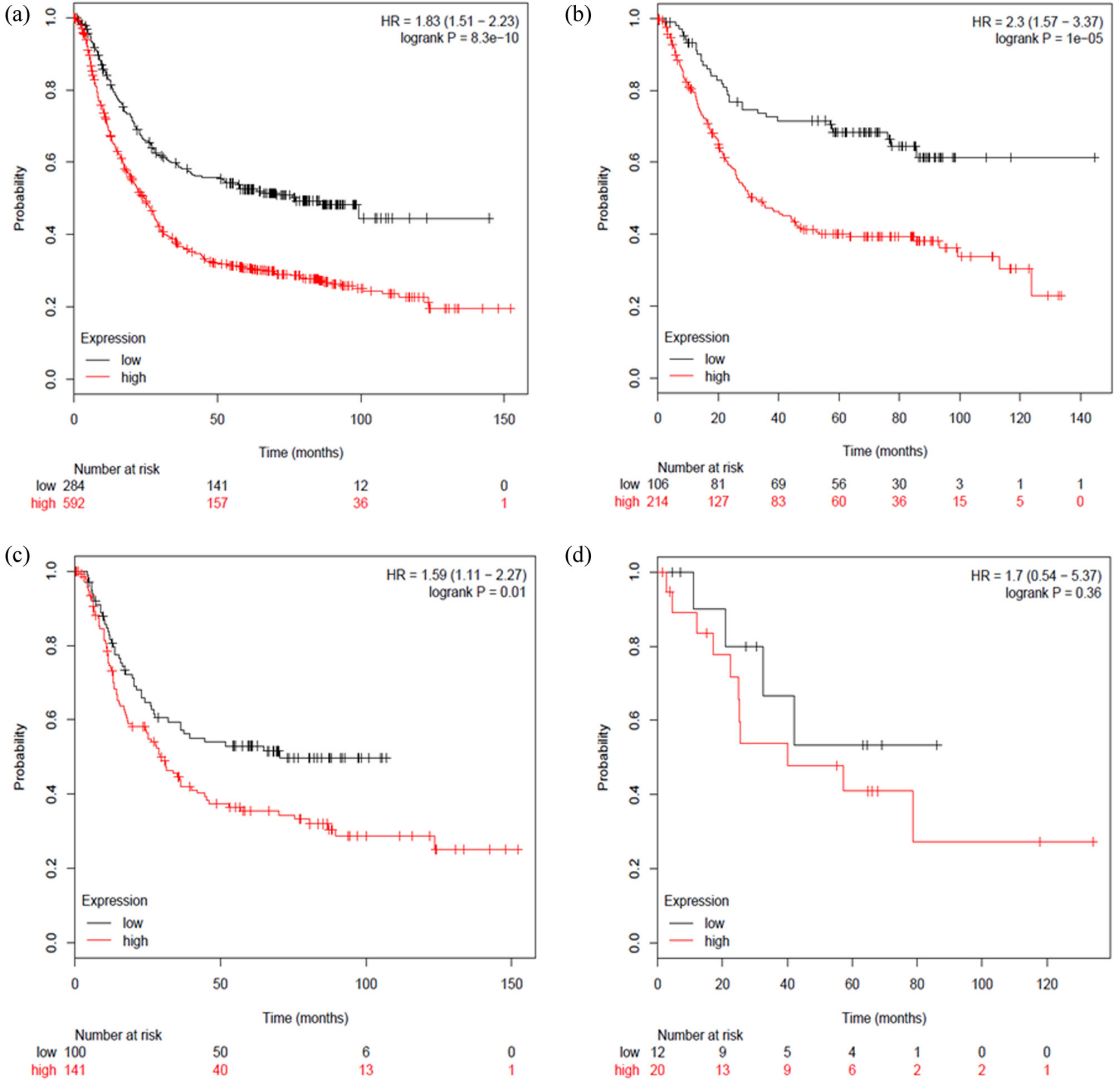
Determination of the prognostic value of RORβ mRNA expression in www.kmplot.com. The desired Affymetrix ID is valid: 206443_at (RORβ). (a) Survival curves were plotted for all patients with GC (*n* = 876). (b) Survival curves were plotted for patients with intestinal-type GC (*n* = 320). (c) Survival curves were plotted for patients with diffuse-type GC (*n* = 241). (d) Survival curves were plotted for patients with mixed-type GC (*n* = 33).

Next, we analyzed the prognostic value of RORβ mRNA expression via www.kmplot.com. As shown in Figure 2, low mRNA expression of RORβ was associated with a better survival [HR = 1.83 95% CI: (1.51–2.23), *P* < 0.0001] (Figure 3a). Low mRNA expression of RORβ was also associated with a better survival in intestinal-type GC [HR = 2.3, 95% CI: (1.57–3.37), *P* < 0.0001] (Figure 3b), as well as in diffuse-type GC [HR = 1.59, 95% CI: (1.11–2.27), *P* = 0.01] (Figure 3c). However, there was not a significant difference in mixed-type GC [HR = 1.7, 95% CI: (0.54–5.37), *P* = 0.36) (Figure 3d).

**Figure 4:**
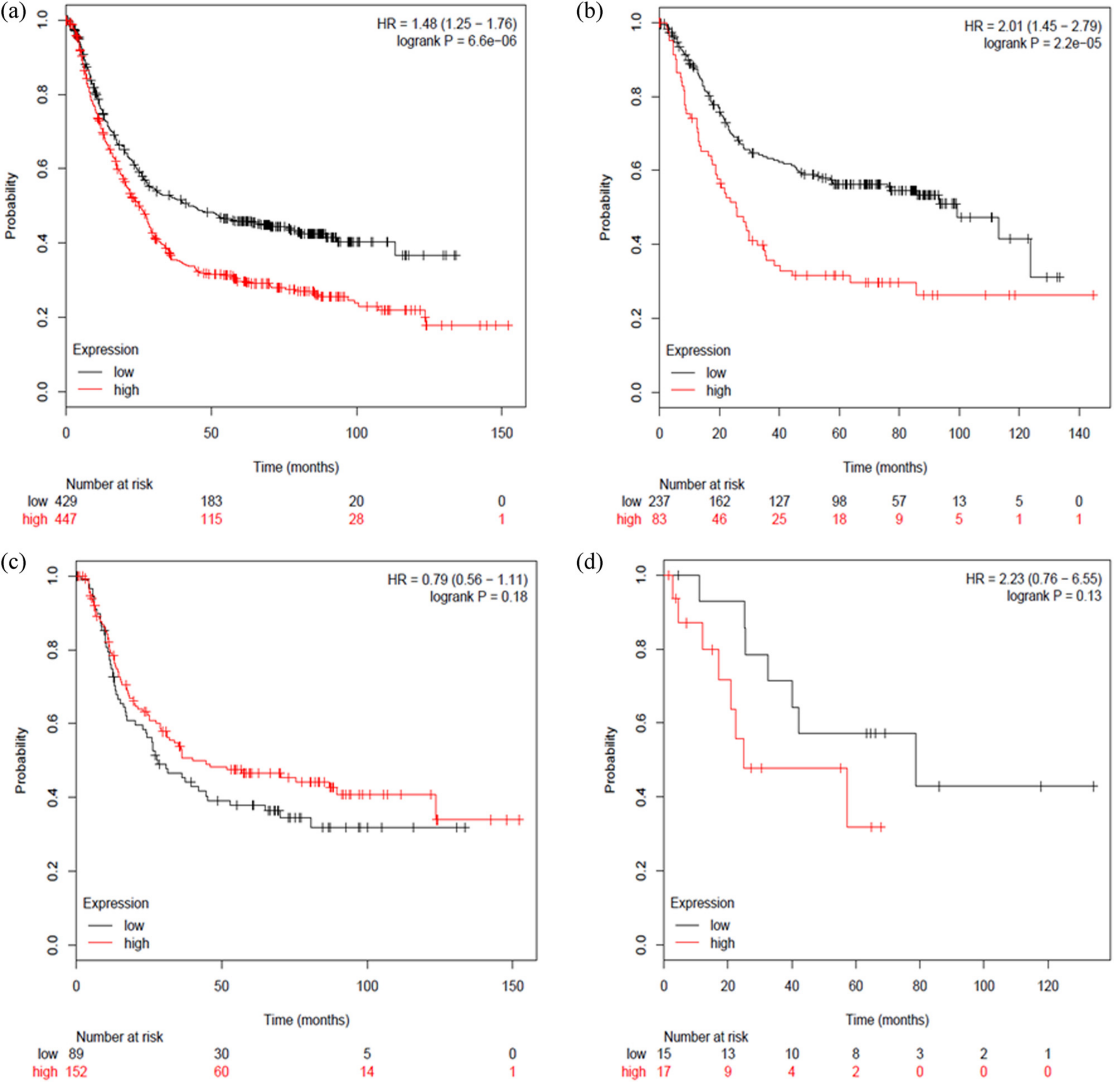
Determination of the prognostic value of RORγ mRNA expression using www.kmplot.com. The desired Affymetrix ID is valid: 206419_at (RORγ). (a) Survival curves were plotted for all patients with GC (*n* = 876). (b) Survival curves were plotted for patients with intestinal-type GC (*n* = 320). (c) Survival curves were plotted for patients with GC (*n* = 241). (d) Survival curves were plotted for patients with mixed-type GC (*n* = 33).

The survival curves of RORγ mRNA expression were shown in Figure 4. We observed that low mRNA expression of RORγ was associated with a better prognosis in GC [HR = 0.48, 95% CI: (1.25–1.76), *P* < 0.0001] (Figure 4a). Low mRNA expression of RORγ was also associated with better prognosis in intestinal-type GC [HR = 2.01, 95% CI: (1.45–2.79), *P* < 0.0001] (Figure 4b). However, there was not a significant difference in diffuse-type GC [HR = 0.79, 95% CI: (0.56–1.11), *P* = 0.18] (Figure 4c) or in mixed-type GC [HR = 2.23, 95% CI: (0.76–6.55), *P* = 0.13)] (Figure 4d).

According to these results, low mRNA expression of RORα, RORβ, and RORγ were all associated with a better prognosis in patients with GC. Although there was not a significant difference in prognosis in the mixed-type GC subgroup, this could be due to the small cohort of only 33 cases. Next, we analyzed the correlation between ROR family members and GC prognosis regarding different clinical pathological features. This included comparing clinical stages (Table 1), HER2 status (Table 2), pathological grades (Table 3), and different treatment methods (Table 4). As shown in Table 1, low RORβ mRNA expression was correlated with a better prognosis in all clinical stages of GC [stage 1: HR = 3.63, 95% CI: (1.35–9.75), *P* = 0.0063; stage 2: HR = 2.71, 95% CI: (1.3–5.67), *P* = 0.0085; stage 3: HR = 1.65, 95% CI: (1.17–2.32), *P* = 0.0036; stage 4: HR = 1.61, 95% CI: (1.1–2.37), *P* = 0.014]. Low mRNA expression of RORα was associated with a better prognosis in clinical stages 2 and 4 in patients with GC [stage 2: HR = 2.13, 95% CI: (1.18–3.86), *P* = 0.01; stage 4: HR = 1.99, 95% CI: (1.37–2.97), *P* = 0.00056]. Low mRNA expression of RORγ was associated with a better prognosis in clinical stages 3 and 4 in patients with GC [stage 3: HR = 2.39, 95% CI: (1.65–3.45), *P* < 0.0001; stage 4: HR = 0.63, 95% CI: (0.42–0.96), *P* = 0.029]. As shown in Table 2, low mRNA expression of RORα [HER2 negative: HR = 1.51, 95% CI: (1.19–1.91), *P* = 0.00064; HER2 positive: HR = 1.36 95% CI: (1.04–1.79), *P* = 0.024] and RORβ [HER2 negative: HR = 1.95, 95% CI: (1.51–2.51), *P* < 0.0001; HER2 positive: HR = 1.59 95% CI: (1.15–2.19), *P* = 0.0046] was associated with a better prognosis in both HER2-negative and HER2-positive patients with GC. In comparison, low mRNA expression of RORγ was only associated with a better prognosis in HER2-negative patients [HR = 1.55, 95% CI: (1.23–1.95), *P* = 0.00016]. As shown in Table 3, the mRNA expression of the different RORs was not correlated with a better prognosis in pathological grade I, II, or III in patients with GC. Finally, as shown in Table 4, low mRNA expression of RORα was associated with a better OS in patients who underwent surgery alone [HR = 2.01, 95% CI: (1.5–2.68), *P* < 0.0001] and in 5 FU-based adjuvant patients [HR = 0.37, 95% CI: (0.24–0.57), *P* < 0.0001]. Low mRNA expression of RORβ was only associated with a better OS in patients who received surgery alone [HR = 1.5, 95% CI: (1.08–2.08), 0.014]. Finally, low mRNA expression of RORγ was only associated with a better OS in 5 FU-based adjuvant patients with GC [HR = 0.69, 95% CI: (0.48–0.97), 0.031].

**Table 1:** Correlation of RORs mRNA expression with clinical stages of patients with GC

ROR	Clinical stages	Cases	HR	95% CI	*P*
RORα	1	69	0.38	0.13–1.14	0.074
	2	145	2.13	1.18–3.86	0.01
	3	319	1.19	0.89–1.59	0.24
	4	152	1.99	1.37–2.97	0.00056

RORβ	1	69	3.63	1.35–9.75	0.0063
	2	145	2.71	1.3–5.67	0.0085
	3	319	1.65	1.17–2.32	0.0036
	4	152	1.61	1.1–2.37	0.014

RORγ	1	69	2.37	0.89–6.34	0.076
	2	145	1.74	0.93–3.26	0.08
	3	319	2.39	1.65–3.45	1.90 × 10^−6^
	4	152	0.63	0.42–0.96	0.029

**Table 2:** Correlation of RORs mRNA expression with HER 2 status of patients with GC

ROR	HER 2 status	Cases	HR	95% CI	*P*
RORα	Negative	641	1.51	1.19–1.91	0.00064
	Positive	425	1.36	1.04–1.79	0.024

RORβ	Negative	641	1.95	1.51–2.51	1.90 × 10^−7^
	Positive	425	1.59	1.15–2.19	0.0046

RORγ	Negative	641	1.55	1.23–1.95	0.00016
	Positive	425	1.26	0.94–1.7	0.12

**Table 3:** Correlation of RORs mRNA expression with pathological grades of patients with GC

ROR	Pathological grades	Cases	HR	95% CI	*P*
RORα	I	166	1.37	0.92–2.04	0.12
	II	67	0.75	0.39–1.45	0.4
	III	32	2.22	0.86–5.75	0.093

RORβ	I	166	0.73	0.49–1.09	0.12
	II	67	0.7	0.35–1.4	0.31
	III	32	0.47	0.16–1.41	0.17

RORγ	I	166	0.81	0.54–1.2	0.29
	II	67	0.51	0.25–1.05	0.063
	III	32	2.6	0.76–8.87	0.11

**Table 4:** Correlation of RORs mRNA expression with different treatment methods of patients with GC

ROR	Treatment	Cases	HR	95% CI	*P*
RORα	Surgery alone	393	2.01	1.5–2.68	1.70 × 10^−6^
	5-FU-based adjuvant	158	0.37	0.24–0.57	3.00 × 10^−6^

RORβ	Surgery alone	393	1.5	1.08–2.08	0.014
	5-FU-based adjuvant	158	1.45	0.99–2.13	0.054

RORγ	Surgery alone	393	1.26	0.94–1.68	0.12
	5-FU-based adjuvant	158	0.69	0.48–0.97	0.031

### 3.2 RORs were upregulated in human GC

**Figure 5:**
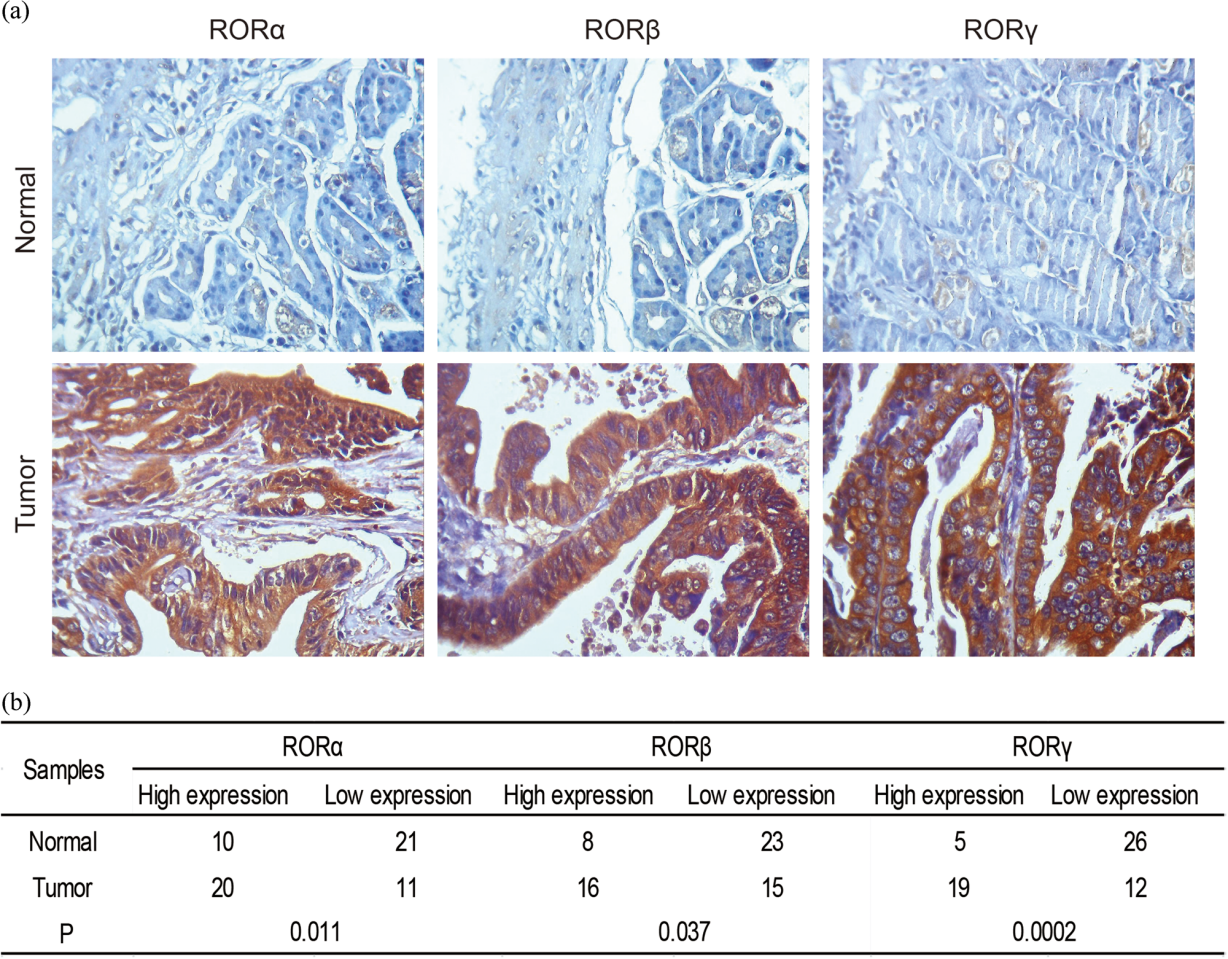
RORα, RORβ, and RORγ were overexpressed in human GC tissues. (a) The expression levels of RORα, RORβ, and RORγ in GC and surrounding tissues were detected by immunohistochemistry. Photomicrographs were taken at 400× magnification. (b) Analysis of immunohistochemical results.

According to our results, high ROR expression presented as a poor prognosis in GC, so we sought to confirm whether RORs were overexpressed in GC tissues. The differentiated expression of RORs in 60 gastric normal tissues and 60 GC tissues of humans was detected using immunohistochemistry. As shown in Figure 5, the expression levels of RORα, RORβ, and RORγ were upregulated in GC tissues when compared with normal tissues. The high expression of RORα, RORβ, and RORγ reached levels of 64.5% (20/31), 51.6% (16/31), and 61.3% (19/31), respectively.

### 3.3 Suppression of ROR expression blocked the proliferation and migration of human GC cells

**Figure 6:**
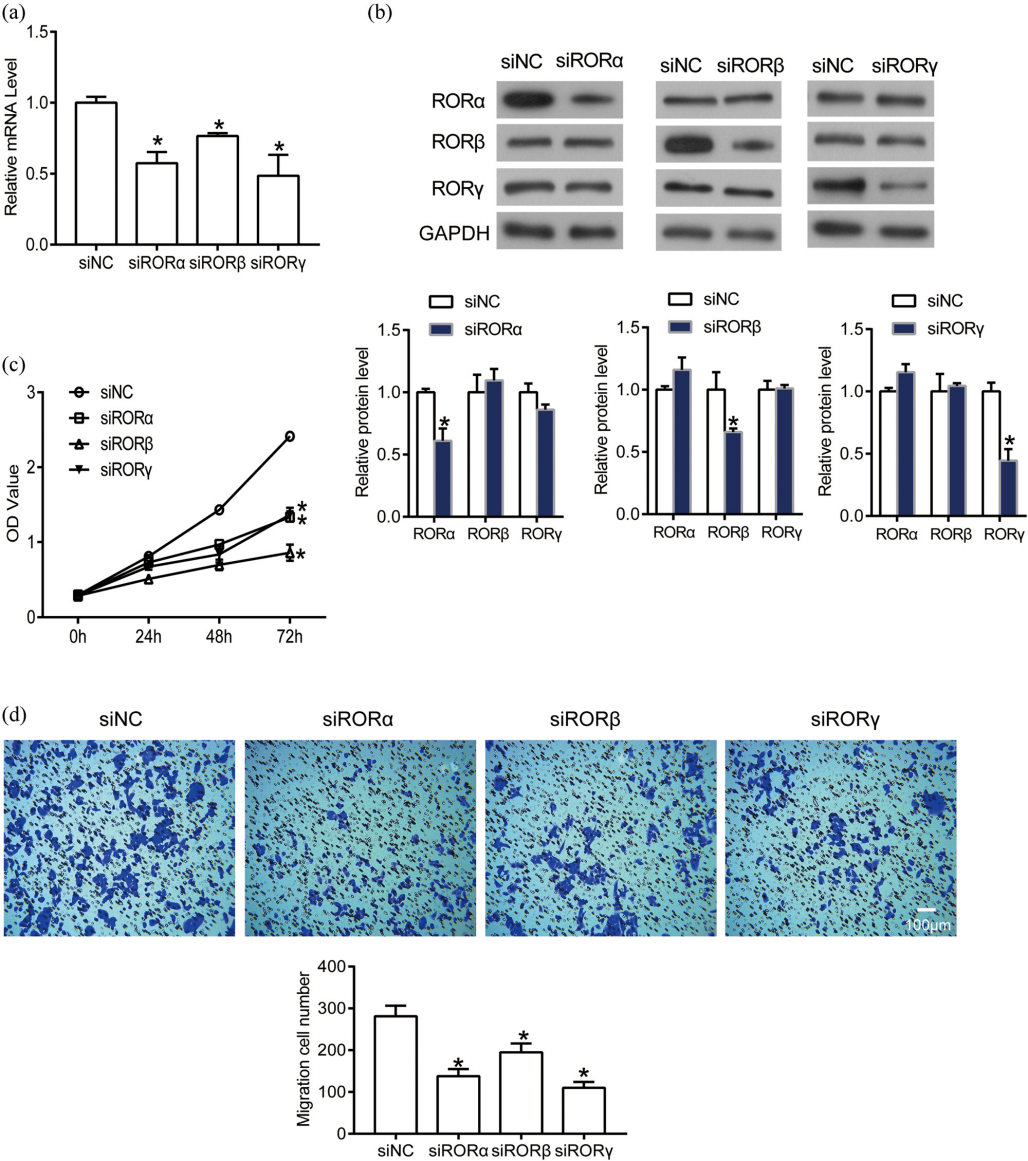
Knockdown of RORα, RORβ, or RORγ inhibited cell proliferation and migration. The mRNA (a) and protein (b) levels of RORα, RORβ, and RORγ in NC and knockdown cells were detected by qPCR and Western blot, respectively. (c) The proliferation of cells with siNC, siRORα, siRORβ, or siRORγ transfection was confirmed by CCK-8 assay. (d) The migration of cells with siNC, siRORα, siRORβ, or siRORγ transfection was detected by transwell. *P < 0.05.

ROR expression was inhibited by specific siRNAs (siRORα, siRORβ, and siRORγ) to build knockdown cells for elucidating their biological functions in AGS cells (Figure 6a and b). We also detected the effect of individual ROR knockdown on other two RORs. The results proved that the expression of single ROR did not affect the expression of other RORs (Figure 6b). Our data showed that following suppression of RORα expression, cell proliferation was significantly inhibited. The same results were also observed in cells in which RORβ or RORγ expression was suppressed (Figure 6c). To further demonstrate the function of RORs in human GC cells, transwell assay was performed. As shown in Figure 6d, the number of migrated cells in siRORα, siRORβ, or siRORγ group was significantly declined when compared with the siNC group. According to the above results, we predict that RORs serve as oncogenes in human GC.

## 4 Discussion

Nuclear receptors are a class of ligand dependent receptors that belong to the transcription factor superfamily, which is similar to steroid hormone receptors. This family is widely distributed in the human body and plays an important role in cell development, cell differentiation, physiological rhythm, metabolism, and immune regulation. Nuclear receptors have three subfamilies consisting of steroid hormone receptors, nonsteroid hormone receptors, and orphan nuclear receptors [18]. RORs are an important members of the orphan nuclear receptor subfamily. They are also named for NR1F due to the homology to the retinoic acid receptor of the steroid receptor family [19].

The RORα gene, located on chromosome 15q22.2, contains 15 exons and spans approximately 730 kb of genomic regions. It has many important physiological functions in organisms. Defects of RORα in mice can lead to a series of abnormalities. These abnormalities can include impaired bone formation and muscle atrophy, ataxia and cerebellar atrophy, decreased size of the spleen and thymus, circadian rhythm disorders, and impaired lipid metabolism [20,21]. Functional analyses have shown that abnormal RORα expression is associated with many diseases and tumor development. RORα can regulate the expression of some tumor related genes (such as nm23 and N-Myc) and can also inhibit tumor development by inhibiting the Wnt/β-catenin signaling transduction pathway [22,23]. In breast tumors, RORα suppresses tumor progression by inducing cell polarization and inhibiting cell invasion, which provides a potential therapeutic target for breast cancer [24]. Colon cancer research has determined that Wnt5a/PKCα-dependent phosphorylation of serine residue 35 of RORα is crucial to link RORα to Wnt/β-catenin signaling. This exerts inhibitory functions of expression of Wnt/β-catenin target genes and provides evidence for a role of RORα between the canonical and the noncanonical Wnt signaling pathways [23]. RORα has also been studied in GC. Positive expression of RORα in gastric carcinoma was significantly lower than matched noncancer mucosa and paracancerous tissue. Furthermore, there was a higher proportion of well-differentiated gastric carcinoma tissue than poorly differentiated gastric carcinoma tissue [25]. However, Bie et al. recently determined that the mRNA expression levels of RORα from patients with GC was higher when compared with healthy controls in peripheral blood mononuclear cells (PBMC) [26]. In our study, low mRNA expression of RORα was correlated with a better prognosis in patients with GC, including intestinal and patients with diffuse-type GC. In addition, RORα can be correlated with prognosis based on different clinical features, including HER2 status, clinical stages 2 and 4, and different treatment methods. Consistent with previous results, RORα was found to be upregulated in tumor tissues and its suppression inhibited cell growth in GC cells. All of the above results suggest that there may be multiple roles of RORα in GC. RORα may be involved in the development of GC by affecting different tumor molecules or signaling pathways.

RORβ was originally identified as a member of the orphan nuclear receptor family by Carlberg in 1994. This gene, located on chromosome 9q21.13, contains 10 exons and spans 188 kb of genomic regions [27]. RORβ plays an important role in the development of many physiological processes and diseases. It can promote the proliferation and differentiation of retinal rod cells, horizontal cells, and amacrine cells [28]. Additionally, it can serve as type of osteogenic inhibitor in the process of bone formation by inhibiting bone differentiation, and it plays an important role in the regulation of physiological rhythm [29,30]. The role of RORβ in tumors has also been studied [31]. Risinger et al. utilized oligonucleotide microarrays to assess the transcript expression profile in epithelial glandular cells. The cells were laser microdissected from 79 endometrioid and 12 serous stage-I endometrial cancers with a heterogeneous distribution of grade and depth of myometrial invasion, along with 12 normal postmenopausal endometrial samples. They found a significantly low expression of RORβ in endometrioid and serous endometrial cancers when compared with a high expression in normal postmenopausal endometrial samples [32]. Interestingly, Davidson et al. demonstrated that the expression level of RORβ was upregulated in primary uterine leiomyosarcoma [33]. Matijevic and Pavelic found that the stimulation of toll-like receptor3 (TLR3) inhibits RORβ expression in pharynx metastatic cell line Detroit 562 and the inhibition of TLR3 results in the upregulation of RORβ. This suggests that RORβ expression is dependent on TLR3 expression [34]. In recent research, RORβ was a key target through which nuclear receptor-interacting protein 2 (NRIP2) regulated activity of the Wnt pathway in enriched colorectal cancer cells. NRIP2 was not able to activate the Wnt pathway after knockdown of RORβ, which suggested that RORβ is also an inhibitor of the Wnt pathway [35]. However, RORβ has not been studied in GC. Though the survival curve, our study determined that low mRNA expression of RORβ was correlated with better prognosis in patients with GC, including intestinal and diffuse subtypes of GC. In addition, RORβ demonstrated correlations based on different clinical features, including HER2 status, clinical stages 1–4, and treatment with surgery alone. Moreover, overexpression of RORβ was observed in GC tissues, and its inhibition blocked the proliferation and migration of AGS cells. Our results indicate that RORβ is associated with the prognosis of GC as an oncogene, but its specific mechanism in GC requires further studies.

The coding gene of RORγ is located on human chromosome 1 and consists of 10 exons. RORγ is mainly expressed in tissues of the immune system, such as the thymus. However, it is also expressed in the liver, skeletal muscle, adipose tissue, and kidney. It plays an important role in the secretion of IL-17 and other proinflammatory factors in Th17 cells [36]. By inhibiting the activity of Th17, RORγ can downregulate the expression of IL-17, maintaining the balance between Th17 and Treg cells, which can reduce the occurrence of inflammatory reactions. RORγ is an important transcription factor that regulates the development and function of CD4-positive Th17 and CD8-positive Tc17 lymphocytes. About 15% of tumors infiltrating lymphocytes express RORγ [37]. In SGC-7901 human GC cells, siRNA-RORγ effectively blocked the expression of SUMO-specific protease 1 (SENP1), hypoxia inducible factor-1α (HIF-1α), and vascular endothelial growth factor (VEGF) production under hypoxia. This indicates the role of nuclear receptor RORγ in melatonin-inhibited HIF-1α and VEGF accumulation [38]. In our study, we determined that low mRNA expression of RORγ was correlated with a better prognosis in GC, including intestinal and diffuse subtypes of GC. In addition, RORγ demonstrated correlations based on different clinical features, including HER2 negative, clinical stages 3 and 4, and 5-FU-based adjuvant treatment. Our results indicate that RORγ is associated with the prognosis of GC. The special role of RORγ makes it a very valuable drug target [39]. Several pharmaceutical companies and research institutions, such as GlaxoSmithKline (GSK) and Scripps Institute, have carried out studies of small molecule ligands targeting RORs [40,41]. Therefore, RORs may promote tumor progression by affecting Wnt and VEGF signaling pathways and the downstream protein expression. In the future, we will focus on the regulation of RORs on the expression of genes related to cell proliferation, metastasis, and apoptosis and the effects on Wnt and VEGF signaling pathways. In addition, considering the interaction between RORs, it is necessary to explore the synergistic effect of two or three RORs. However, due to the limitations of online analysis, we need to collect clinical organizations in local hospitals for research, which will also be the focus of our further research.

In conclusion, expression levels of RORα, RORβ, and RORγ were all correlated with the prognosis of GC. Low mRNA expression of RORs indicated a better OS based on the existing pathological samples. RORs were upregulated in GC tissues and their knockdown inhibited the proliferation and migration of GC cells. Therefore, it is necessary to expand the sample size to study the prognostic value of RORs and to study the specific pathways of RORs and their roles *in vivo*. These findings suggest that all three ROR subfamilies play an important role in the development of GC and have potential as anticancer supplements. This provides new ideas and approaches for the treatment of GC.
